# About a case: affective psychosis and hyperthyroidism

**DOI:** 10.1192/j.eurpsy.2023.1625

**Published:** 2023-07-19

**Authors:** J. Peñalver, A. Llimona, J. Mayans, L. N. Vargas, M. T. Campillo, C. Muro, S. Oller

**Affiliations:** 1 Psiquiatría, Parque de Salut Mar; 2 Psiquiatria, Parc de Salut Mar; 3 Psiquiatria, Parque de Salut Mar, Barcelona, Spain

## Abstract

**Introduction:**

Hyperthyroidism due to Graves-Basedow disease is a common cause of neuropsychiatric manifestations, such as anxiety, psychomotor restlessness, mood disturbances, insomnia and psychosis. Hashimoto’s encephalopathy rarely occurs in so-called autoimmune thyroiditis, which can present with hyperthyroidism and neuropsychiatric symptoms similar to Graves’ disease. We add that the mystical-religious beliefs, present in all human cultures, and decisive in the case at hand, make us propose an evolutionary origin of them.

**Objectives:**

Clinical case description

**Methods:**

A clinical case based on medical reports is described

**Results:**

We present the case of a 72-year-old woman, a member of the Seventh-day Adventist Church, well adapted to the Community. Known history of elevated antithyroid antibodies since 2019, brought to the emergency room involuntarily due to a mystical-religious delusional condition associated with behavioral disturbance. On examination, cachectic appearance, distal tremor, emotional exaltation and megalomanic speech were highlighted. Laboratory tests revealed primary hyperthyroidism with elevated antibodies. During admission, the differential diagnosis between Graves-Basedow disease and Hashimoto’s encephalopathy was considered. Thyroid scintigraphy oriented the diagnosis to Graves-Basedow disease, not requiring lumbar puncture or corticosteroid treatment. Treatment was based on high-dose antithyroid and antipsychotic drugs, with clinical and analytical remission at 3 weeks. The patient was referred to a Social Health Center for functional recovery. The family refers to a similar episode in 2014, of less intensity and self-limited, which is proposed to be a hashitoxicosis.

**Conclusions:**

Differential diagnosis between Graves-Basedow and Hashimoto disease is essential as they differ in treatment and prognosis. The continuity that the delusion presents with the previous beliefs of the patient, differing mainly in the affective-behavioral implication, makes us consider a predisposition to psychosis in our patient. Religiosity can be adaptive in certain environments, since mystical beliefs have existed throughout the history of the human species and seem to be part of our nature.

**Disclosure of Interest:**

None Declared

